# Radiosurgery: Teenage Sex or Midlife Crisis?

**DOI:** 10.3389/fsurg.2022.875111

**Published:** 2022-03-25

**Authors:** Manjul Tripathi

**Affiliations:** Department of Neurosurgery, Postgraduate Institute of Medical Education and Research, Chandigarh, India

**Keywords:** radiosurgery (SRS), gamma knife ®, quality of life, minimally invasive, CyberKnife

## Abstract

Martin Luther King, in his famous speech, mentioned that “Blood alone moves the wheels of history. Stereotactic radiosurgery (SRS) moved the wheel of neurosurgery in the absence of bloodshed. Over the last five decades, SRS has made a phenomenal stride in the pursuit of being minimally invasive but equally effective. Though literature testifies for its effectiveness, feasibility, and applicability, the traditional mindset of a neurosurgeon feels it difficult to accept it open heartedly. Radiosurgery is essentially a neurosurgeon's tool with more partial, conservative, and pragmatic approach with sole intention to maintain lesion control while preserving the quality of life of the patient. It demands a thorough knowledge to be impregnated into young neurosurgeon's mind at the time of their training, else it would fascinate or frighten with biased opinions.

“In my early fifties, I was going through a phase where few things felt right, and I was trying to figure out those that did. It was not uncommon. In your twenties, you pursue your dreams. By your late thirties and early forties, you hit a certain stride. Then you hit your fifties, you get your first annoying thoughts of mortality, you begin more serious questioning of not just the meaning of your life but of what's working, what's not working, and what you still want, and all of a sudden you don't know which way is up. You thought you knew but don't. You just want to get to where life feels okay again.”

— Dick Van Dyke, “My Lucky Life in and Out of Show Business”

Munching on the history of Neurosurgery, I realized that neurosurgery took a sharp turn in the 1980s. The introduction and development of micro neurosurgery by Gazi Yasargil made a sea change in the philosophy of neurosurgery. Suddenly, alpha neurosurgeons started considering themselves invincible. They attacked each nook and corner of the human brain, and anything deemed alien to the brain was considered worthy of being taken out. The arachnoid web transformed from a sinewy trap to a guiding pathway that drained CSF, allowing fissures to open-up, and guide surgeons as they slid seamlessly into the depths of the brain. Everything looked promising, too promising to be true. Much water has passed under the bridge from the enucleation of vestibular schwannoma to intraoperative neuronavigation and planned partial resection. However, we are still far from being invincible. Bearing the same frustration, Lars Leksell took a quick walk-in solitude during the matinee session of a conference. His thinking mind was perplexed with the miseries of neurosurgical endeavors, and he came up with the idea of stereotactic radiosurgery (SRS) (1).

Neurosurgeons love the drama inside the “operation theatre” and the persona of a neurosurgeon (2). Most centers have terrific, pragmatic, fast, and smart neurosurgeons. They are confident and tough guys. But, we occasionally find patients marginally improved or even worse than their preoperative status. Neurosurgeons looked down on SRS as a radiation tool and were hostile to neurosurgical philosophy up to three decades earlier. How could some radiation treat a benign or vascular pathology? It seemed a dull and overzealous attempt by those neurosurgeons who had little interest in surgery. But somehow, it maintained its stand and increased its reach to most of the neurosurgical ailments.

For a beginner, radiosurgery seems as fascinating as teenage sex. It has all the ingredients of adolescent attraction. An early career neurosurgeon feels pretty excited and loves to try it. Looking at the limited radio surgical centers in teaching institutes, radiosurgery appears to be a mystical and novel first experience with the radiation tool in the realm of a neurosurgeon. However, without proper guidance, the initial attempts do not feel that enjoyable as there is no immediate result, no blood bath, and the surgeon needs to wait for a longer time for the lesion to disappear or primarily reduce by some quarters at its best. Until you understand the philosophy of radiosurgery, you are bombarded with conflicting messages from traditional neurosurgical practitioners and competing radiation oncologists. As you no more battle with blood and flesh, you remain concerned about what peers think about you. Neurosurgeons interested in radiosurgery feel threatened to be labeled “chicken hearted” or non-operative neurosurgeons; however, once you understand it and learn the science and craft of radiosurgery, you want to perform much better every time you do it.

On the other hand, SRS proved to be a distracting force for some seasoned neurosurgeons. SRS especially made a paradigm shift in managing two intracranial disorders; vestibular schwannoma and arteriovenous malformation (3, 4). It is not uncommon to hear a debate in current neurosurgical conferences on micro neurosurgery vs. radiosurgery for many intracranial diseases. This changed the surgical practice of many traditional skull base and vascular neurosurgeons. With this sweeping change, a hostile environment emerged, mainly because of neurosurgeons trained before the popularity of radiosurgery. They perceived the gamma knife as a threat to the surgical knife and an unwanted encroachment in their field. With growing literature supporting the effectiveness of this new blade, a state of midlife crisis has started to settle in the neurosurgical mindset, where the surgeon is married to the operating room but is having an affair with the radiosurgery station.

Neurosurgery seems far more dynamic in present times (5, 6). In the current era, radiosurgery is an integral aspect of the neurosurgical armamentarium. It is an established field of neurosurgical practice flourishing and prospering with great participation of neurosurgeons themselves. Radiosurgery remains a viable alternative for many neurosurgical patients, and statistics show that patients are happy with the radio surgical experience. With the ever and rapidly growing literature in support of SRS, the stakes have significantly changed. The focus is now on maintaining a good quality of life with long-term lesion control. Once you start understanding operative neurosurgery's limitations and safety profile, you start thinking of radiosurgery as a helpful adjunct or sometimes stand-alone therapy. As you gain practice and experience, coming out of this midlife crisis, you tend to look at the art behind the science. When you are a Picasso or a Rembrandt, the difference is no longer of skill but one of artistic choice. An expert neurosurgeon has to choose between cure and control for their patient, and the question of which knife to use and how remains a philosophical question and Sophie's choice.

## Author Contributions

The author confirms being the sole contributor of this work and has approved it for publication.

## Conflict of Interest

The author declares that the research was conducted in the absence of any commercial or financial relationships that could be construed as a potential conflict of interest.

## Publisher's Note

All claims expressed in this article are solely those of the authors and do not necessarily represent those of their affiliated organizations, or those of the publisher, the editors and the reviewers. Any product that may be evaluated in this article, or claim that may be made by its manufacturer, is not guaranteed or endorsed by the publisher.
